# A Mildly Acidic Environment Alters Pseudomonas aeruginosa Virulence and Causes Remodeling of the Bacterial Surface

**DOI:** 10.1128/spectrum.04832-22

**Published:** 2023-06-06

**Authors:** Negar Mozaheb, Paria Rasouli, Mandeep Kaur, Patrick Van Der Smissen, Gerald Larrouy-Maumus, Marie-Paule Mingeot-Leclercq

**Affiliations:** a Université catholique de Louvain, Louvain Drug Research Institute, Cellular & Molecular Pharmacology Unit (FACM), Brussels, Belgium; b Université catholique de Louvain, de Duve Institute, CELL Unit and PICT Platform, Brussels, Belgium; c Imperial College London, Department of Life Sciences, MRC Centre for Molecular Bacteriology and Infection, Faculty of Natural Science, London, United Kingdom; University of Nebraska Medical Center

**Keywords:** *Pseudomonas aeruginosa*, envelope, membrane vesicles, acidic pH, low pH

## Abstract

Pseudomonas aeruginosa is a versatile pathogen that resists environmental stress, such as suboptimal pH. As a result of exposure to environmental stress, P. aeruginosa shows an altered virulence-related phenotype. This study investigated the modifications that P. aeruginosa undertakes at a mildly low pH (pH 5.0) compared with the bacteria grown in a neutral medium (pH 7.2). Results indicated that in a mildly acidic environment, expression of two-component system genes (*phoP*/*phoQ* and *pmrA*/*pmrB*), lipid A remodeling genes such as *arnT* and *pagP* and virulence genes, i.e., *pqsE* and *rhlA*, were induced. Moreover, lipid A of the bacteria grown at a mildly low pH is modified by adding 4-amino-arabinose (l-Ara4N). Additionally, the production of virulence factors such as rhamnolipid, alginate, and membrane vesicles is significantly higher in a mildly low-pH environment than in a neutral medium. Interestingly, at a mildly low pH, P. aeruginosa produces a thicker biofilm with higher biofilm biomass. Furthermore, studies on inner membrane viscosity and permeability showed that a mildly low pH causes a decrease in the inner membrane permeability and increases its viscosity. Besides, despite the importance of PhoP, PhoQ, PmrA, and PmrB in Gram-negative bacteria for responding to low pH stress, we observed that the absence of each of these two-component systems does not meaningfully impact the remodeling of the P. aeruginosa envelope. Given that P. aeruginosa is likely to encounter mildly acidic environments during infection in its host, the alterations that the bacterium undertakes under such conditions must be considered in designing antibacterial strategies against P. aeruginosa.

**IMPORTANCE** P. aeruginosa encounters environments with acidic pH when establishing infections in hosts. The bacterium develops an altered phenotype to tolerate a moderate decrease in the environmental pH. At the level of the bacterial envelope, modified lipid A composition and a reduction of the bacterial inner membrane permeability and fluidity are among the changes P. aeruginosa undergoes at a mildly low pH. Also, the bacterium is more likely to form biofilm in a mildly acidic environment. Overall, these alterations in the P. aeruginosa phenotype put obstacles in the way of antibacterial activities. Thus, considering physiological changes in the bacterium at low pH helps design and implement antimicrobial approaches against this hostile microorganism.

## INTRODUCTION

Pseudomonas aeruginosa is an opportunistic pathogen that resists various stresses, such as the presence of antibacterial agents, nutrient deprivation, and an acidic pH. The significance of the changes that P. aeruginosa undergoes when grown in stressful environments is highlighted when these changes synergistically impact the decrease in bacterial susceptibility to antibacterials (1).

During infection, P. aeruginosa encounters an environment with an acidic pH. The microenvironment of the P. aeruginosa biofilm is mildly acidic (between pH 4 and 6), likely due to environmental DNA (eDNA) (2). Additionally, the airways of cystic fibrosis (CF) patients and the urinary tract provide pH 6, which can be infected by P. aeruginosa (3, 4). This bacterium shows a different phenotype when residing in an acidic environment from that in a neutral pH (5).

At highly acidic pH (pH below 4), the proton accumulation in the bacterial cells massively damages macromolecules, and thus, various chaperones are induced. In particular, due to low pH, unfolded proteins and perturbation of bacterial envelope integrity activate the heat shock response factor (σ^70^) and envelope stress response factor (σ^22^) or AlgU, respectively (6, 7). Additionally, the cellular SOS response is activated at low pH to maintain the structure and function of the bacterial genomic material and guarantee bacterial survival and propagation (8). At a mildly acidic pH, the presence of protons does not damage cell macromolecules, yet it disrupts cellular energy production via the proton motive force (PMF) (9). Indeed, the electrochemical proton gradient across the cell membrane (Δ*P*) and membrane potential (ΔΨ) contribute to the generation of PMF. Given that PMF is the driving force for ATP synthesis, the bacteria tightly avoid the dissipation of PMF (10). Thus, P. aeruginosa applies various approaches to resist mildly acidic environments. Among those is changing the bacterial envelope composition to decrease the interaction of the bacterial surface with protons accumulated outside the cell (9, 11).

In Gram-negative bacteria, the envelope consists of inner and outer membranes. Lipopolysaccharide (LPS) is the main component of the outer membrane, which comprises three distinct domains, the O-antigen, the core region, and lipid A. The O-antigen consists of repeating units of sugar residues. The core region has 3-deoxy-ulosonic acid (Kdo), linked to a short chain of sugar residues on one side. Another side is directly linked to lipid A. Lipid A is comprised of a β-1′,6-linked disaccharide of glucosamine that is phosphorylated and acylated. This structure is conserved among Gram-negative bacteria. However, it undergoes various alterations to optimize bacterial adaptation to the environment (12, 13). Overall, the changes that cell membranes go through to fulfill their dedicated functions in biological processes are called “membrane remodeling.” In Gram-negative bacteria, membrane remodeling occurs at the inner and outer membranes (14).

A mildly acidic environment induces two two-component systems (TCSs) in P. aeruginosa, namely, PhoP/PhoQ and PmrA/ PmrB (2). The sensory kinases of these systems, PhoQ and PmrB, are transmembrane histidine kinases (15), which are autophosphorylated at low pH and transfer the phosphate to the intracellular parts PhoP and PmrA, respectively (16). PhoP and PmrA are transcription factors that identify promoter regions of various operons related to lipid A modification genes. In particular, 4-amino-arabinose (l-Ara4N) transferase (ArnT) (17), lipid A palmitoyl transferase (PagP) (18), and lipid A deacylase (PagL) (19) play pronounced roles in modifying lipid A upon induction of the two TCSs. In addition to lipid A modification genes, these two TCSs can regulate the expression of virulence factors (20).

Outer membrane modification at low pH can lead to the generation of lipid A species having a high tendency to bleb from the outer membrane and form membrane vesicles (MVs) (21, 22). Additionally, in P. aeruginosa, induction of the membrane stress response pathway via activation of AlgU positively regulates membrane vesiculation (23, 24). Moreover, the bacteria can exploit MVs to increase the bacterial membrane turnover rate and accelerate the development of the adapted LPS according to the environment (22). Thus, the bacteria utilize MV production to gain environmental adaptation.

In addition to alterations that P. aeruginosa outer membrane undertakes upon encountering stressors, the bacterial inner membrane could provide the bacterium with a higher adaptability to low-pH challenges. Altered membrane viscosity and permeability are among the modifications made to the bacterial inner membrane in response to low pH stress to reduce proton passage across the cell membrane (11).

Moreover, upon exposure to stress, P. aeruginosa produces Pseudomonas quinolone signal (PQS) (25) and extracellular polysaccharides, such as Pel, Psl, and alginate (26, 27). These virulence factors increase bacterial surface hydrophobicity, the likelihood of self-aggregation, and surface attachment to help in biofilm development (5). Also, in P. aeruginosa, *pqsE* codes the final protein in the PQS biosynthesis operon (*pqsABCDE*), and PqsE positively regulates the expression of P. aeruginosa virulence factors, such as *rhlA* (28). *rhlA* is a critical gene in the rhamnolipid production cassette (29). Stress-induced rhamnolipid production provides P. aeruginosa with increased protection against insults (30, 31).

The present study explored P. aeruginosa's fitness for coping with mildly acidic pH. We assessed the impact of growing at a mildly low pH on the production of virulence-related factors in P. aeruginosa and the biophysiochemical characteristics of the bacterial envelope. We comparatively analyzed the expression of genes contributing to altering lipid A composition and the production of virulence factors by P. aeruginosa PAO1 at neutral (pH 7.2) versus mildly acidic (pH 5.0). Additionally, we assessed the possible modification of lipid A and the rate of membrane vesiculation at mildly low pH and in the absence of the sensory kinases (PmrB and PhoQ), and we compared them with those in the neutral medium. Furthermore, we studied the alterations of P. aeruginosa’s inner membrane viscosity, permeability, and membrane potential at a mildly low pH.

## RESULTS

### Growing at a mildly low pH causes a noticeable delay in P. aeruginosa growth.

**FIG 1:**
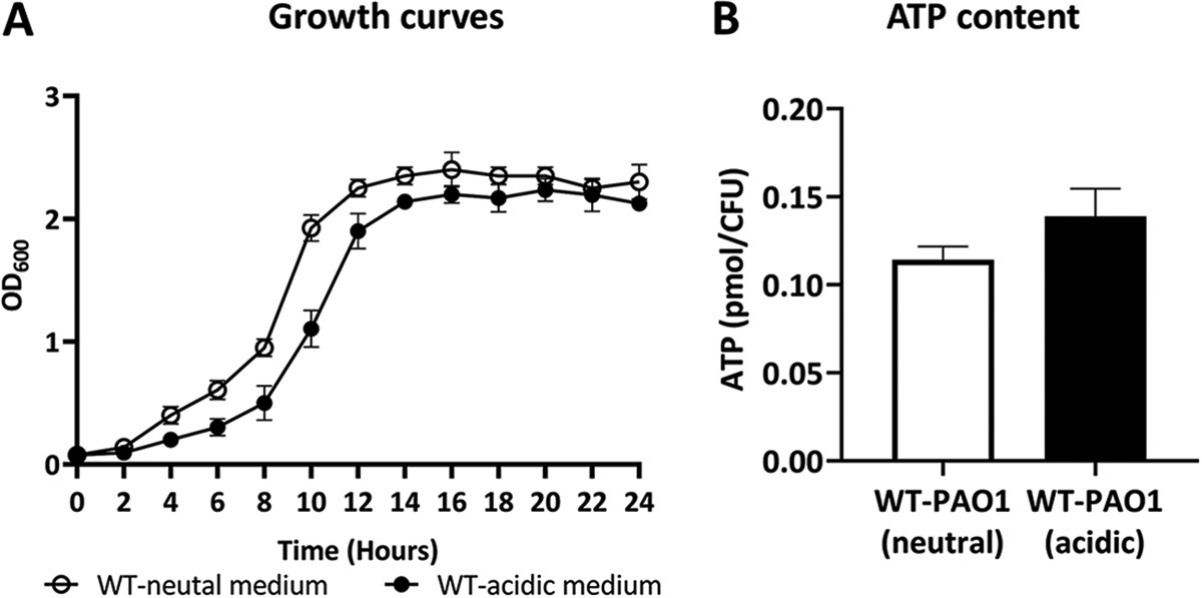
Growth curve and ATP analysis. (A) Growth curves of strains over 24 h in neutral (pH 7.2) and acidic (pH 5.0) LB. (B) ATP content of the bacteria. Data are presented as mean ± SD. Statistical analysis was performed on two biological replicates and three technical replicates via multiple *t* tests.

To understand the effect of a mildly acidic environment on the fitness of P. aeruginosa, we compared the growth rates of the bacteria and their doubling times. Our observation showed a delay in the growth of P. aeruginosa in the mildly acidic medium (pH 5.0) in comparison with their counterparts in the neutral medium (pH 7.2) (Fig. 1A). The comparative doubling time study showed that in the mildly acidic medium, the doubling time of P. aeruginosa is 49.6 ± 1.1 min, approximately 11% longer than that of the bacteria in the neutral medium (44.3 ± 6.2 min). However, the result of the ATP assay showed no significant difference in the bacterial ATP content in the neutral versus acidic medium (Fig. 1B).

### A mildly low pH alters gene expression and production of virulence factors in P. aeruginosa.

**FIG 2:**
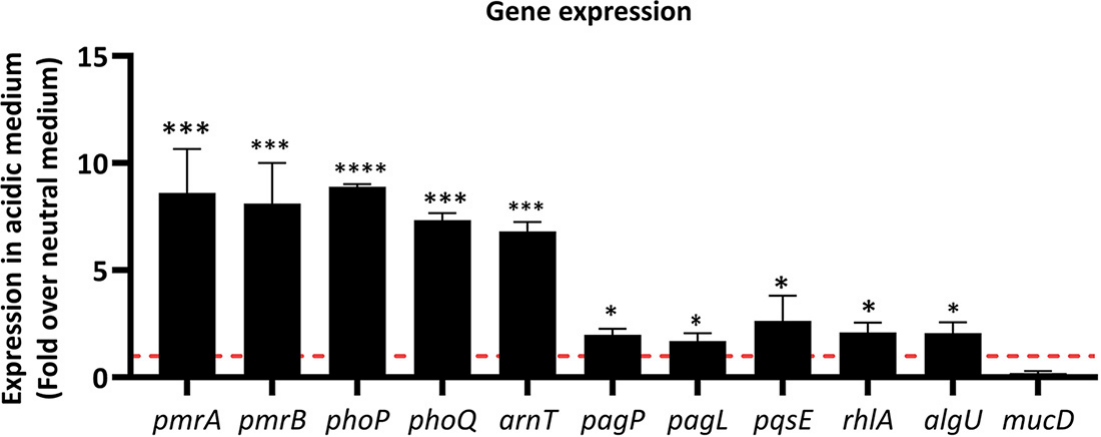
Gene expression. Relative gene expression analysis of P. aeruginosa in a mildly acidic medium. The comparison is relative to the expression in the control group (cells in the neutral medium). The red dashed line is showing one representing the gene expression in the control group, and the housekeeping 16S rRNA gene was used as the internal control. Data presented as mean ± SD. Statistical analysis was performed on two biological and two technical replicates via multiple *t* tests, ****, *P < *0.0001; ***, *P < *0.001; *, *P < *0.05.

The comparative gene expression analysis showed significant overexpression of the genes coding for sensor kinases (*pmrB* and *phoQ*) and the transcription factors (*pmrA* and *phoP*) in the mildly acidic medium in comparison with that in the neutral pH. Among the genes coding the lipid A modification enzymes, there was a roughly 8-fold increase in the expression level of *arnT* in the mildly acidic medium. Additionally, *pagP* and *pagL* were expressed slightly higher in the mildly acidic medium. Also, in the acidic medium, a small but significant enhancement was observed in the transcripts of various genes related to the bacterial virulence factors, such as *pqsE* and *rhlA*. Moreover, quantitative PCR (qPCR) results showed overexpression of the envelope stress transcription factor (*algU*), which can be related to the slightly mucoid appearance of the bacterial colonies at a mildly acidic pH. Nonetheless, there was no significant difference in the expression of the AlgU inhibitor (*mucD*) (Fig. 2).

**FIG 3:**
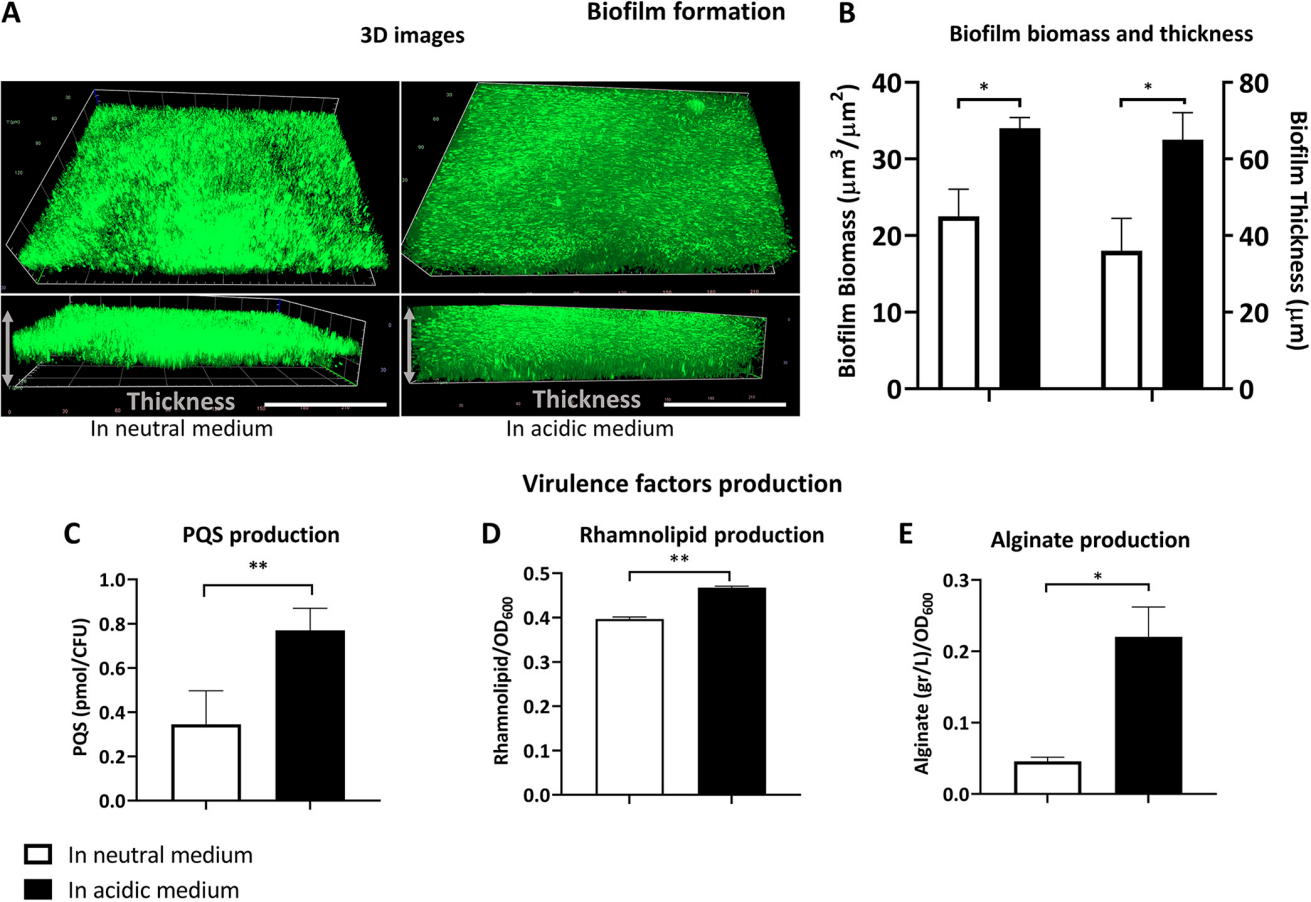
Biofilm, PQS, rhamnolipid, and alginate production. (A) Three-dimensional (3D) image of the P. aeruginosa biofilms (stained with SYTO 9) formed in the neutral (left) and the mildly acidic media (right). The scale bars represent 100 μm. (B) Comparison of the biofilm biomass and the biofilm thickness. The calculations were performed on values obtained from two independent biological replicates and at least two technical replicates. (C to E) Comparative quantification of PQS (C), rhamnolipid (D), and alginate (E) production. Data are presented as mean ± SD. Statistical analysis was performed on three biological replicates via multiple *t* tests; **, *P < *0.01; *, *P < *0.05.

The induction of virulence genes expression in the mildly acidic medium is not only at the level of the gene transcription but also leads to the formation of thicker biofilm with a larger amount of biofilm biomass (Fig. 3A and B). Also, in the mildly acidic medium, P. aeruginosa overproduced some virulence factors such as PQS (Fig. 3C), rhamnolipid (Fig. 3D), and alginate (Fig. 3E).

### At a mildly low pH, P. aeruginosa modifies its lipid A.

**FIG 4:**
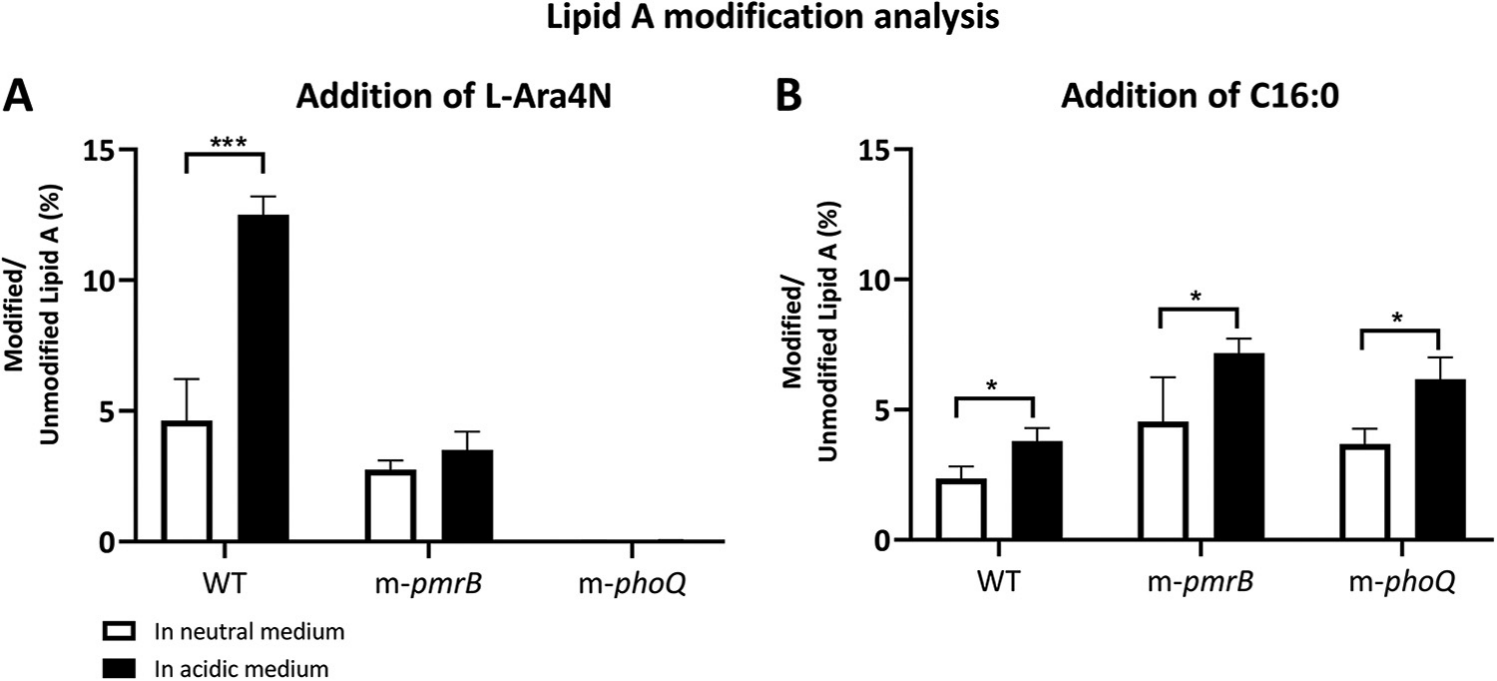
Lipid A modification analysis. Comparison of lipid A modified with l-Ara4N (A) and C_16:0_ (B). The comparison has been made based on the most abundant lipid A species (penta- and hexa-acylated lipid A at *m/z* 1,447 and 1,616). Data are presented as mean ± SD obtained from two independent biological replicates. Multiple *t* tests were utilized for statistical analysis. ***, *P < *0.001; *, *P < *0.05.

In continuation of exploring P. aeruginosa alterations at a mildly low pH, we investigated the modifications of lipid A. In P. aeruginosa, changes in the fatty acid composition of lipid A by adding l-Ara4N and palmitate (C_16:0_) are among the bacterial surface modifications in an acidic environment, and they are found in the clinical isolates (32). Thus, we compared the proportion of the lipid A modified with the addition of l-Ara4N (Fig. 4A) and C_16:0_ (Fig. 4B) relative to the unmodified lipid A species in the neutral and acidic media. Additionally, we explored the changes in the percentage of modified lipid A species in P. aeruginosa PAO1 transposon insertion mutants, m-*pmrB* or m-*phoQ*. The lipid A analysis showed that at neutral pH, wild-type (WT) P. aeruginosa PAO1 and m-*pmrB* possessed approximately less than 5% of lipid A species modified with l-Ara4N. In the mildly acidic medium, the percentage of modified lipid A with l-Ara4N in the WT strain reached 12%, roughly 2-fold higher than that in the neutral pH. Interestingly, although no differences were observed at neutral pH in lipid A modified with l-Ara4N between WT and m-*pmrB*, at acidic pH, this modification was 3-fold lower in m-*pmrB* than that in the parental strain. In the m-*phoQ* strain, the percentage of lipid A species modified with l-Ara4N was marginal and considerably less than that in the parental strain (Fig. 4A). Moreover, as seen in Fig. 4B, acidic pH leads to a nearly 1.5-fold increase in lipid A that contains an extra C_16:0_ in WT, m-*pmrB*, and m-*phoQ*.

Additionally, due to the function of PagL, an increase in the proportion of penta- to hexa-acylated lipid A can be expected. In contrast, we observed a noticeable accumulation of hexa-acylated lipid A, including the C_10:0_ acyl chain, in strains grown in the acidic medium compared to those in the neutral medium (Fig. S1A). It is worth noting that we studied the MIC of polymyxin B and colistin against P. aeruginosa. The result showed that the difference in MIC between the strains at neutral and acidic pH is marginal (Table S1).

### Growing in the mildly acidic medium, P. aeruginosa produces a higher quantity of MVs.

**FIG 5:**
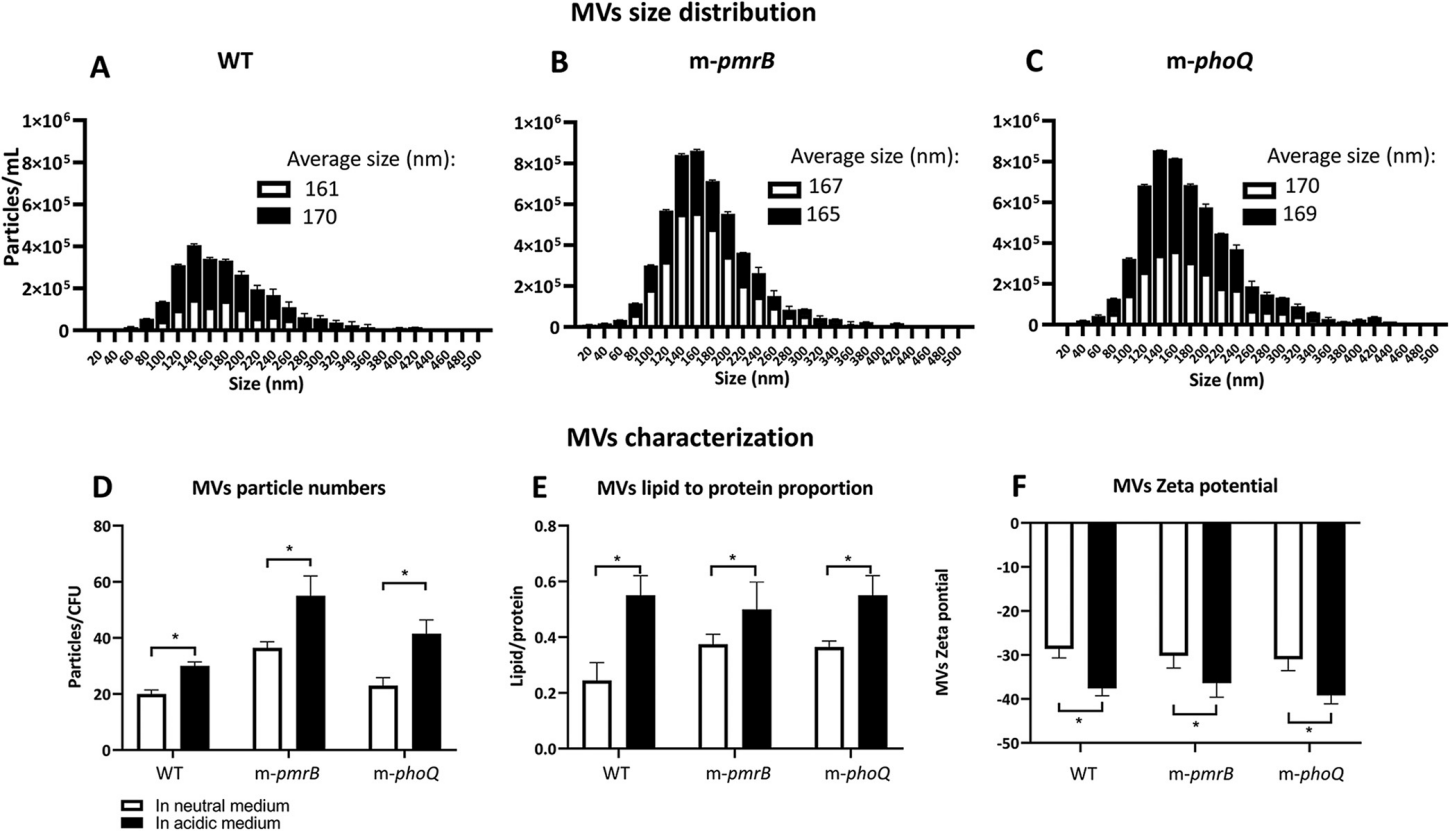
MV quantification analysis. (A to C) Size distribution of the MVs produced by WT, m-*pmrB*, and m-*phoQ*, respectively. (D) Particles of MVs normalized by CFU numbers. (E) Lipid-to-protein proportion of the MVs. (F) MV zeta potential. The data are presented as mean ± SD from two biological and two technical replicates under each condition. Statistical analysis was performed by multiple *t* tests. *, *P < *0.05.

Exposure to stress and the difference in lipid A composition could lead to differences in MV quantity and changes in MV characteristics. We investigated the MV size distribution, particle numbers, relative lipid-to-protein composition, and their surface charge (zeta potential). Results showed that the MVs produced by WT, m-*pmrB*, and *m*-phoQ at neutral or mildly acidic pH had similar size distributions (Fig. 5A to C). Additionally, in the mildly acidic medium, all studied strains produced a remarkably higher number of MVs per CFU (Fig. 5D). The MVs produced in the mildly acidic medium had a significantly greater proportion of total lipids to proteins (Fig. 5E). Also, the zeta potential analysis revealed that the mildly acidic pH caused the production of MVs with a significantly higher surface negative charge than those produced in the neutral pH (Fig. 5F). These observations revealed that low pH not only changes the composition of the bacterial surface (lipid A) but also leads to alteration of the bacterial MV composition. The lipid A analysis of MVs confirmed the differences in the MV lipid A composition in the mildly acidic medium from that in the neutral medium (Fig. S1B).

### The mildly acidic environment increases P. aeruginosa’s inner membrane viscosity and decreases the inner membrane permeability.

**FIG 6:**
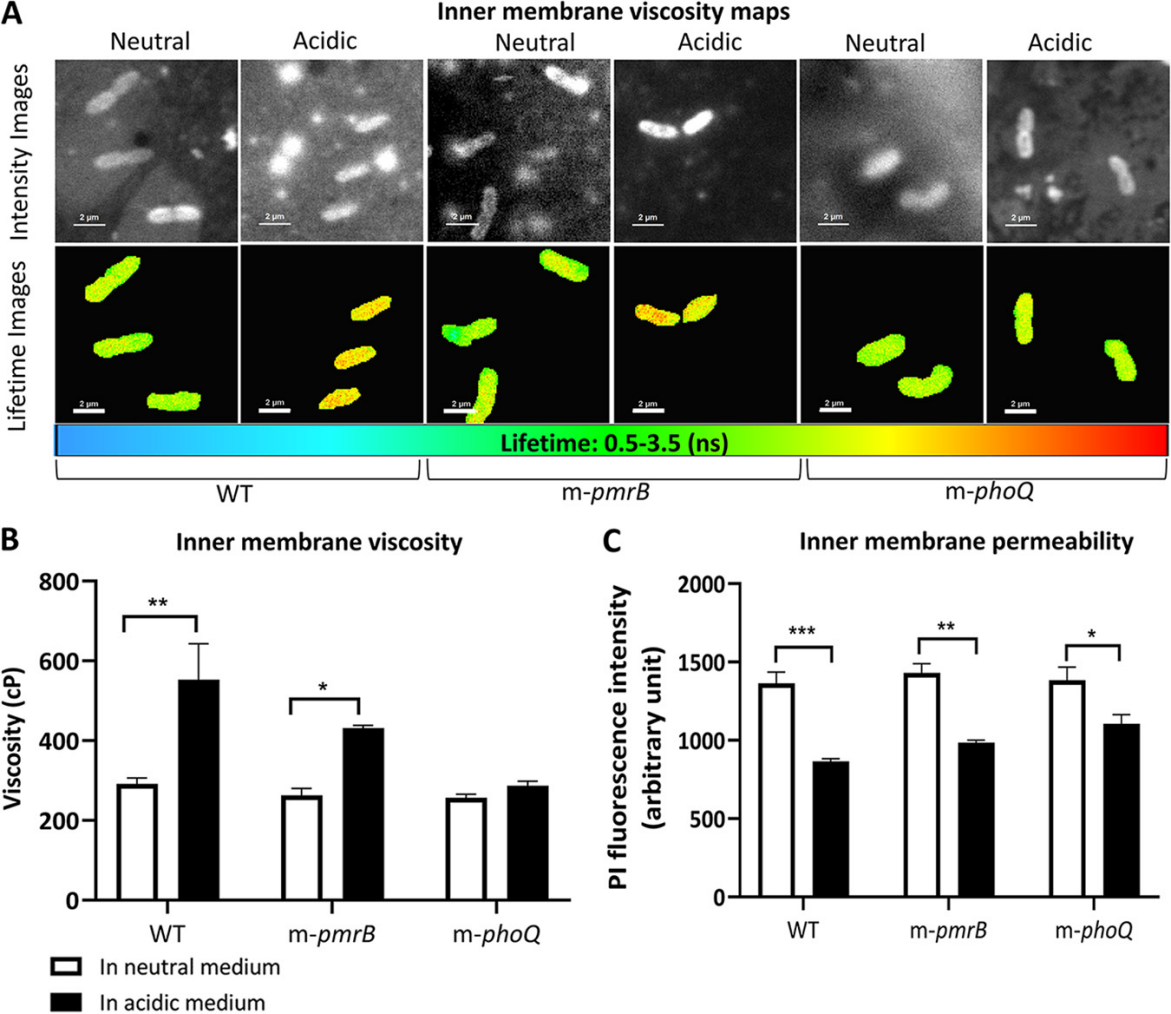
Inner membrane viscosity and permeability study. (A) FLIM observation for comparing the bacterial membrane viscosity labeled with BODIPY-C10. (A, Top) Intensity images. (A, Bottom) Lifetime images. Scale bars represent 2 μm. (B) Comparative viscosity of the bacterial membranes according to the probe’s lifetime. The data were obtained from two individual experiments, and in each experiment, at least 5 bacteria or three slides were considered to measure the lifetimes of the bacteria. (C) Comparison of the bacterial inner membrane permeability based on the intensity of PI accumulated inside the cells. The data are presented as mean ± SD from two biological and at least three technical replicates under each condition. Statistical analysis was performed by multiple *t* tests. ***, *P < *0.001; **, *P < *0.001; *, *P < *0.01.

To explore the alterations of P. aeruginosa’s inner membrane grown in the mildly acidic medium, we assessed the membrane viscosity and permeability via fluorescence lifetime imaging microscopy (FLIM) and the propidium iodide (PI) accumulation assay, respectively (Fig. 6). FLIM observations demonstrated that growing at a mildly low pH resulted in an increase in the bacterial membrane viscosity (Fig. 6A and B). PI has marginal diffusion across the intact membrane of P. aeruginosa. However, environmental conditions and membrane composition could alter the bacterial membrane permeability to PI. The inner membrane permeability assay revealed that in the acidic medium, the intensities of PI accumulated inside the WT, m-*pmrB*, and m-*phoQ* cells were, respectively, 35%, 30%, and 20% less than those of their counterparts in the neutral medium (Fig. 6C). This result indicates that at a mildly low pH, the bacterial inner membrane is less permeable to the diffusion of PI. Also, m-*phoQ* at low pH showed a higher permeability to PI than the WT at the same condition.

### A mildly low pH does not dissipate P. aeruginosa membrane potential.

**FIG 7:**
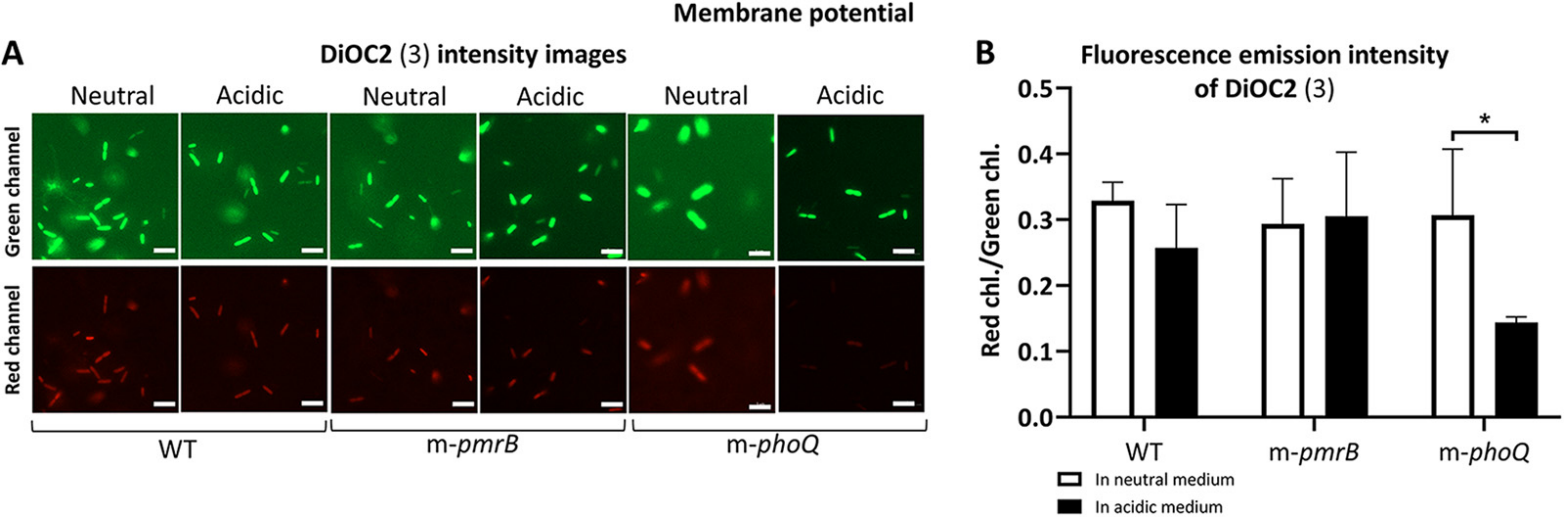
Membrane potential analysis via DiOC_2_(3) intensity assay. (A) The fluorescence emission of the probe incorporated into the bacterial membranes is shown in the green channel (top panels) and the red channel (bottom panels). Scale bars represent 5 μm. (B) Ratio of the fluorescence intensity in the red to the green channel. Data representing the mean values ± SD, obtained from three biological replicates. Statistical analysis was performed using multiple *t* tests. *, *P < *0.05.

We investigated the bacterial bioenergetic state via a fluorescent probe to further explore the effect of growing at a mildly acidic pH on the bacterial surface. We questioned whether a mildly low pH decreases membrane polarization and dissipates the bioenergetic process in the cells. We assessed the emission of the cells labeled with DiOC_2_(3) in the red and green channels. A higher proportion of red-to-green emission intensity shows more polarized membranes and energized cells. Results showed that the red-to-green emission decreased marginally in the WT strain when the bacteria were grown in a mildly acidic pH compared to that in the neutral medium. Moreover, there was no significant difference in the membrane potential of m-*pmrB* at neutral and mildly acidic conditions. Nevertheless, when the *m*-phoQ was grown in the mildly acidic medium, a significant reduction was observed in the red-to-green emission proportion compared to that in the neutral medium. This result showed that pH 5.0 caused the dissipation of membrane polarity in the m-*phoQ* strain (Fig. 7A and B). Additionally, we observed a considerable delay in m-*phoQ* growth time in the mildly acidic medium compared to that in the neutral medium and the WT strain (Fig. S2).

## DISCUSSION

**FIG 8:**
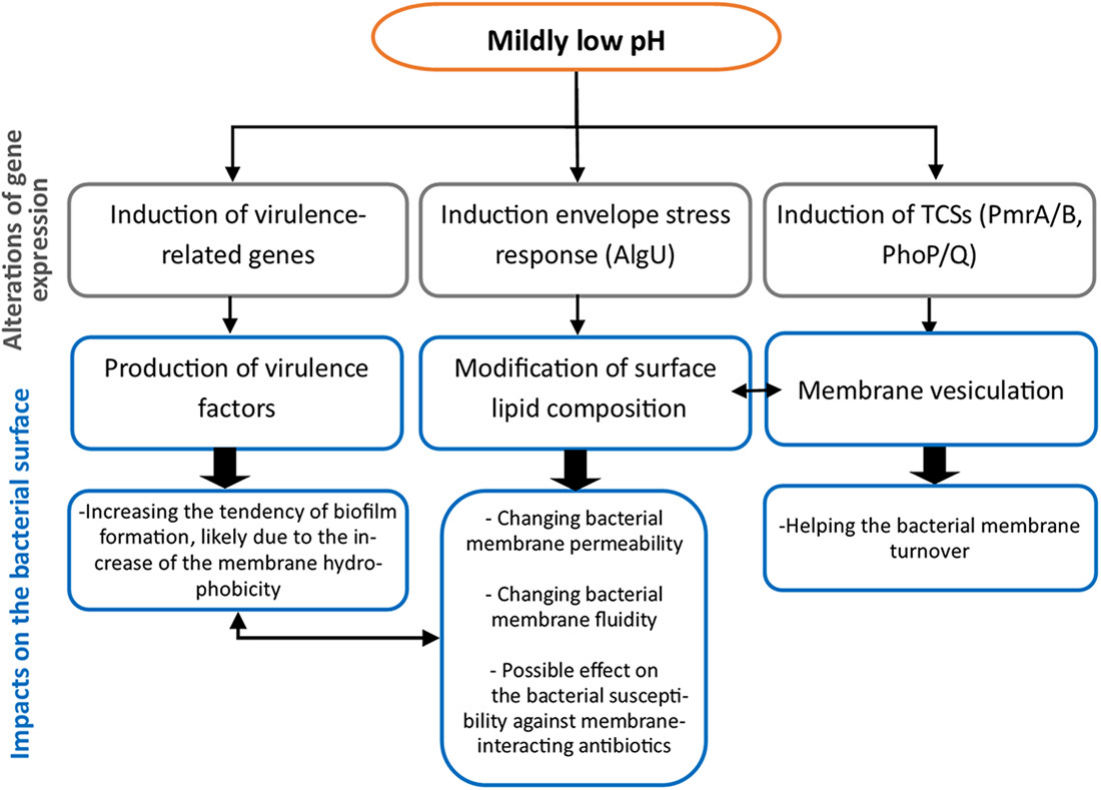
Schematic overview of alteration of P. aeruginosa at a mildly low pH.

There is accumulating evidence that P. aeruginosa is more virulent in an acidic environment than in a neutral medium (5, 9, 11). Our study supports this emerging hypothesis and indicates that the responses of P. aeruginosa in a mildly acidic medium can exacerbate the virulence characteristics of the bacterium. The schematic view of possible alterations in P. aeruginosa at a mildly low pH is illustrated in Fig. 8.

A slightly low pH can stress P. aeruginosa, and the bacterial growth rate and energy molecule (ATP) production can be impaired under these conditions. Therefore, this stress induces bacterial surface changes (10). Our observations showed that although there was a notable delay in the growth of P. aeruginosa in the mildly acidic medium, the cells were likely to have roughly similar amounts of ATP in the mildly acidic and neutral media. The bacterial growth rate and ATP content are a trade-off between the presence of nutrition in the environment and the energy cost of producing biomolecules (33). Therefore, in this study, the delay in the growth rate can be caused by the overexpression of virulence factors, which puts a metabolic burden on the bacteria. However, the advantages of overproducing some virulence factors remain to be determined (34, 35).

Stress-induced responses in P. aeruginosa could cause the development of modified phenotypes resistant to a particular group of antimicrobials. P. aeruginosa not only senses low pH (2), but it also perceives Mg^2+^ deficiency (16) and the presence of polycationic antibiotics and antimicrobial peptides (36) via the two TCSs (PmrA/B and PhoP/Q). Thus, environmental stressors lead to the evolution of a cross-resistant population of the bacterium against the “last hope” antibiotic, i.e., polymyxins (2, 36).

The comparative study of the gene expressions in the mildly acidic medium compared to those in the neutral medium revealed significant overexpression of *pmrB* and *phoQ* and transcription factors (*pmrA* and *phoP*) of the TCSs (37). *pmrA*/*B* and *phoP*/*Q* operons regulate the expression of *arnBCADTEF* operon, *pagP*, and *pagL*, responsible for adding l-Ara4N (38) to lipid A, palmitoylation, and deacylation of lipid A (39), respectively. In this study, we observed that a mildly low pH induces expression of *arnT*, *pagP*, and *pagL*. Also, microarray studies of P. aeruginosa revealed that numerous virulence genes possess regulatory sequences, which PmrA and PhoP identify (16). Thus, they could upregulate the expression of the virulence genes. In particular, we observed significant overexpression of *pqsE*, *rhlA*, and *algU*, crucial genes for producing virulence factors such as PQS, rhamnolipid, and alginate, respectively. Additionally, these virulence factors regulate the expression of genes contributing to biofilm formation in P. aeruginosa (26, 30, 40). Of note, at mildly acidic conditions, the *pmrA*/*B* and *phoP*/*Q* loci are subjected to autoregulation (2, 37, 41). Hence, it is not surprising that the overexpression of the TCS elements is noticeable. Nonetheless, the expression of other genes (*pagP*, *pagL*, *pqsE*, *rhlA*, and *algU*) that belong to different operons can be affected by intricate and interconnected gene expression regulators in P. aeruginosa. Thus, the increase in the transcription levels of *pagP*, *pagL*, *pqsE*, *rhlA*, and *algU* was to a smaller extent than that of the elements of TCSs.

The comparative lipid A analysis confirmed a significant enhancement of lipid A species modified with the addition of l-Ara4N and C_16:0_ at a mildly low pH, which agrees with the qPCR result regarding the overexpression of *arnT* and *pagP*, respectively. PmrA and PhoP upregulate the expression of *arnT*, and in the absence of PhoQ, *pmrA* is upregulated, and it can compensate for the lack of PhoP/Q (42). In P. aeruginosa, these TCSs are more likely to work convergently. Thus, the TCSs may compensate for the absence of each other in regulating lipid A modification. Previously, it was shown that deletion of *phoQ* in polymyxin-resistant P. aeruginosa results in a gain of function of PmrB and overexpression of the *arnBCADTEF* operon (43). PagL removes 3-hydroxydecanoate (3OH-C_10:0_) from hexa-acylated lipid A to generate penta-acylated lipid A. Thus, an increased presence of penta-acylated compared to hexa-acylated was expected in the acidic medium. Additionally, a study on Salmonella showed the importance of PagL for membrane vesiculation in an acidic environment. Vesiculation helps the bacteria remodel their membrane (22).

The variation between the comparative gene expression data related to lipid A modification enzymes and the abundance of modified lipid A motivated us to compare the quantity and composition of the MVs produced by the wild type and mutants of P. aeruginosa in the neutral medium with those produced in the mildly acidic medium. The origins of the MVs and their contribution to lipid A remodeling have yet to be fully understood. However, evidence shows that their production helps with bacterial surface remodeling. Moreover, alterations in the composition of bacterial membrane lipids can lead to the production of MVs (44). This study showed low pH induces membrane vesiculation, and those MVs are shown to have a higher lipid-to-protein ratio. Indeed, lipid A variations lead to membrane vesiculation. In particular, lipid A palmitoylation is known to be effective in the induction of MV production (21). Also, vesiculation accelerates the acquisition of an adapted lipid A species by bacteria via increasing the rate of membrane exchanges (45). This observation agrees with the lipid A analysis of the MVs, as in the acidic medium, no lipid A modified with l-Ara4N was detected (see Fig. S1B in the supplemental material). It supports the hypothesis that adapted lipid A species to the environment are less likely to be incorporated into MVs (21, 22). In addition to the changes in the lipid A composition in the mildly acidic medium that could lead to membrane vesiculation, activation of the membrane stress response-transcription factor (AlgU) in the mildly acidic environment could positively regulate membrane vesiculation (23).

Further, we studied P. aeruginosa’s inner membrane viscosity and permeability at a mildly low pH. Previous studies revealed the increase of bacterial outer membrane viscosity due to the induction of PmrA/B and PhoP/Q followed by lipid A modification (46). In this study, we observed an increase in inner membrane viscosity at a mildly low pH using the FLIM technique and a fluorescent probe labeling the bacterial inner membrane (47, 48). Indeed, in Gram-negative bacteria, the inner membrane is the crucial barrier to controlling proton influx (11). Studies showed that at low pH, an enhanced proportion of hopanoids (bacteriohopanepolyols) in the inner membrane of the Gram-negative bacteria reduces the diffusion of protons inside the cells. Moreover, the presence of hopanoids increases the viscosity of the bacterial membrane (11, 49). Regarding the biophysical characteristics of the m-*phoQ* membrane (inner membrane permeability, viscosity, and membrane potential), we observed noticeable differences between the mutant and the parental strains in both neutral and acidic media. These observations are aligned with a previous study on the regulatory role of PhoP/Q in changing the glycerophospholipid composition of the bacterial membrane (50). However, further studies have to be done on the contribution of PhoP/Q to changing the composition of the P. aeruginosa membrane. Additionally, the adaptability response of P. aeruginosa at low pH, by adding l-Ara4N to lipid A, potentially serves as an essential coping strategy for P. aeruginosa (20).

### Conclusion.

This study aimed to explore the physiological changes that P. aeruginosa undergoes in a mildly acidic environment. P. aeruginosa expresses a more virulent phenotype in an ecosystem with a mildly low pH than in a neutral pH environment via the production of virulence factors and by changing the biophysicochemical characteristics of its surface. Considering that P. aeruginosa is likely to encounter low ambient pH during infection, this exacerbates the pathogenicity of P. aeruginosa and can hinder the antibacterial effects against this pathogen. In this study, for the first time, we investigated the genotypic and phenotypic changes of P. aeruginosa together with outer membrane composition and vesiculation under a mildly low pH. Also, we showed the alterations of inner membrane viscosity and permeability in a mildly acidic environment. Thus, the study helps better understand the pathophysiology of this versatile microorganism.

## MATERIALS AND METHODS

### Medium preparation and bacterial strains.

For making the neutral medium, LB medium was prepared (Miller’s modification; Merck), and the pH was adjusted to 7.2 using 3-(*N*-morpholino) propane sulfonic acid (MOPS buffer). For the acidic medium, the pH was adjusted to 5.0, using a 2-(*N*-morpholino) ethanesulfonic acid (MES) buffer. The m-*pmrB* and m-*phoQ* mutant derivatives of P. aeruginosa PAO1 were acquired from the transposon mutant two-allele library (University of Washington, Seattle, WA; Manoil laboratory) (51, 52). The transposon mutants were confirmed by colony PCR according to the method described in the supplemental material and using the primers in Table S2.

### Growth curve and doubling time.

Overnight culture of P. aeruginosa PAO1 was suspended in LB (neutral and acidic) at 10^5^ CFU/mL concentrations. For determining the growth curves, the bacteria were incubated at 37°C with shaking at 180 rpm, and the optical densities at 600 nm (OD_600_) of the bacterial cultures were measured every 2 h.

### ATP quantification.

The ATP concentration of the bacterial cells was assessed using the BacTiter-Glo microbial cell viability assay (Promega). At the end of the logarithmic phase of growth, the bacterial cell numbers were adjusted to ~10^7^ CFU and subjected to an ATP assay.

### Biofilm preparation and imaging.

In 24-well polystyrene plates with a coverslip at the bottom, the biofilms in the neutral and the acidic LB were prepared. The planktonic bacteria were removed after 20 h, and the biofilms were rinsed with phosphate-buffered saline (PBS) and dyed with SYTO 9. After 15 min, the leftover dye molecules were removed, and the coverslips were washed. The coverslips were placed and viewed using a cell observation spinning disk microscope (Carl Zeiss) with an oil immersion 40× objective. SYTO 9 was detected in the green channel (excitation and emission of 488 and 502 to 538 nm, respectively), and the images were obtained in the z-stack scanning mode at a resolution of 1,388 by 1,040 pixels. ZEN 2.6 (blue edition) software was used to create the three-dimensional graphics. COMSTAT 2.1 was used to measure the biomass and biofilm thickness (53).

### RNA extraction and qPCR.

After 12 h (at the end of the logarithmic growth phase), the cells were pelleted by centrifugation at 2,978 × *g* for 20 min at 4°C (Eppendorf 5810 R centrifuge; A-4-62 rotor). RNA was extracted from bacterial cells (~10^9^ CFU) using an RNA extraction kit (Invitek), and the extracted RNAs were treated with DNase (Turbo DNA-free kit) prior to cDNA synthesis. Using SYBR green supermix (Bio-Rad) and particular primers for *pmrA*, *pmrB*, *phoP*, *phoQ*, *arnT*, *pagP*, *pagL*, *pqsE*, *rhlA*, *algU*, and *mucD* genes, quantitative PCR (qPCR) was conducted (Table S3). The relative levels of gene expression were calculated via the threshold cycle (ΔΔ*C_T_*) method, and 16S rRNA was used as a control (54).

### Rhamnolipid extraction and quantification.

Rhamnolipids were extracted and quantified using a previously described protocol (55). The cells were pelleted via centrifugation under the conditions outlined above. We used 8 mL of diethyl ether to extract rhamnolipids from 1 mL of the bacterial supernatants. The organic was evaporated at room temperature, the precipitant was dissolved in water (100 μL), and 900 μL of a mixture containing 0.19% (wt/vol) orcinol in 50% H_2_SO_4_ was added to it. Samples were heated at 80°C for 10 min and cooled to room temperature for 10 min. The absorbance was measured at 421 nm. The OD_600_ of the bacterial culture before rhamnolipid extraction was used to calculate the normalized data.

### Alginate extraction and quantification.

Alginates were extracted according to the formerly described method (56). Briefly, alginates were precipitated by mixing the bacterial supernatants (10 mL) with 2% cetylpyridinium chloride (20 mL). Alginates were then obtained by centrifugation (10,000 × *g* for 10 min at room temperature) and resuspended in 500 mL of isopropanol. After an hour of incubation at −20°C, alginate pellets were centrifuged at 10,000 × *g* for 10 min at 4°C and then resuspended in 500 mL of 1 M NaCl. For alginate quantification, 50 μL of the extracted alginate was mixed with 200 μL of a 25-mM sodium tetraborate/sulfuric acid reagent (2 M H_3_BO_3_ in sulfuric acid). The mixture was then heated to 100°C for 10 min and cooled at room temperature for 15 min. Then, 50 μL of 0.125% carbazole reagent (in 100% ethanol) was added to the mixture. The mixture was then heated to 100°C for an additional 10 min. After cooling for 15 min, the concentration of alginates was measured according to the absorbance of the solution and standard curve of alginic acid at 550 nm.

### PQS extraction and quantification.

PQS was extracted from 10 mL of culture supernatant of P. aeruginosa using 10 mL acidified ethyl acetate (0.01% acetic acid) (57). The organic phase was transferred to a new tube, and the solvent was evaporated via a nitrogen stream. A normal-phase silica 60 F_254_ high-performance thin-layer chromatography (HPTLC) plate was soaked in KH_2_PO_4_ (5%) and activated at 100°C for 1 h. The extracted PQS was dissolved in methanol and spotted on the HPTLC plates. The plate was placed in the HPTLC chamber presaturated with the mobile phase (2-propanol/ethyl acetate [4:6]). The commercial PQS (2-heptyl-3-hydroxy-4-quinolone; Merck) was used as a positive control and for quantifying the PQS produced by P. aeruginosa. The densitometry of the applied PQS spots was performed on a Camag TLC scanner at 254 nm (58).

### MV isolation and purification.

At the end of the logarithmic phase, bacterial cells cultured in one-fifth of the volume of an Erlenmeyer flask were removed by centrifuging at 2,978 × *g* for 20 min at 4°C to isolate MVs from planktonic bacteria (Eppendorf 5810 R centrifuge; A-4-62 rotor). The supernatant was centrifuged at 150,000 × *g* for 3 h at 4°C through a 0.45-μm polyvinyl difluoride (PVDF) filter (Whatman) (Beckman; 80 Ti rotor). The supernatant was discarded, and the pellet was resuspended in an MV buffer containing 10 mM HEPES and 0.85% NaCl (pH 7.2). Isolated MVs were purified further using a gradient density of OptiPrep-iodixanol (Sigma-Aldrich) in MV buffer, as previously described (59, 60). ZetaVIEW S/N 18-400 was used to do the MV size analysis. A previously described protocol was used to measure the lipid content and lipid-to-protein ratio (61). Briefly, the MVs were labeled with a lipophilic fluorescence dye (FM 4-64 dye) at 5 g/mL. ZetaVIEW S/N 18-400 was used to do the MV size analysis. The MVs were labeled with a lipophilic fluorescence dye (FM 4-64 dye) to measure the lipid content at 5 g/mL (SpectraMax M3). Increasing amounts of water-soluble linoleic acid (Sigma-Aldrich; product no. L5900) and 5 g/mL FM 4-64 dye were used to create a calibration curve. The protein content of the MVs was determined using the Pierce bicinchoninic acid (BCA) protein assay kit.

### Lipid A study.

Lipid A from P. aeruginosa was extracted with the MBT Lipid Xtract kit (Bruker Daltonics, Germany), following the manufacturer’s instructions. Briefly, bacteria were grown overnight in Mueller-Hinton agar, and the equivalent of a 1-μL inoculation loop was placed into a 1.5-mL low-binding microtube and mixed in 50 μL of MTB Lipid Xtract hydrolysis buffer. We discarded 44 μL of the cell suspension, and the remaining 6 μL was submitted to a heating process at 90°C for 10 min. The tubes were left for 2 min with the lid open to completely evaporate the buffer. The dried pellets were washed with 50 μL of washing buffer without dissolving the pellet. The total volume of the washing buffer was discarded by pipetting. Finally, 5 μL of the matrix was pipetted up and down for 15 to 20 s to resuspend the dried pellet, and 2 μL was spotted onto an MSP 96 polished steel BC target (Bruker; part no. 8280800). The bacterial suspension and matrix were mixed directly on the target by pipetting and then dried gently under a stream of air. The spectra were recorded in the linear negative-ion mode (laser intensity, 45%; ion source 1, 15.00 kV). Each spectrum corresponded to an ion accumulation of 200 to 1,000 laser shots randomly distributed on the spot. The spectra obtained were processed with default parameters using FlexAnalysis v.3.4 software (Bruker Daltonik, Germany).

### FLIM slide preparation.

The protocol for the preparation of bacterial slides was adapted from a previous study with modifications (47, 48). Briefly, 10^7^ CFU/mL of cells were suspended in PBS containing BODIPY-10 (0.5 μM) and glucose (0.1%) at a concentration of 10^7^ CFU/mL, followed by incubating them at 37°C with 180 rpm shaking for 1 h. Then, 200 μL of the cell suspensions was subsequently immobilized on the 8-well ibidi microscopy chamber, which had been precoated with 0.1% poly-l-lysine.

### FLIM imaging.

Bacteria were observed on the LSM980 multiphoton microscope (Zeiss, Germany) equipped with a time-correlated single-photon-counting (TCSPC) FLIM module (PicoQuant, Germany) for high-resolution microscopy. A Coherent (Chameleon Discovery) pulsed laser (80 MHz) at 800 nm was used to excite BODIPY-C10. The emission was captured with a 505- to 545-nm bandpass filter at a resolution of 512 by 512 pixels. The lifetime images were obtained by recording fluorescence lifetimes in each pixel of the image corresponding to the bacteria. The lifetime values (τ) ranged from 0.5 to 3.5 ns and were shown with blue (short) to red (long) pseudocolor code.

### FLIM analysis.

The FLIM images were analyzed using the SymPhoTime64 software (PicoQuant, Germany). Using the viscosity-lifetime calibration equation 1, which was established by measuring the fluorescence lifetime (τ) of BODIPY-C10 in several methanol-glycerol mixtures with known viscosities, the lifetime data were converted into viscosity (η) data (48).

(1) $$\log\;\eta =\frac{\text{log τ}+0.75614}{0.4569}$$

### Inner membrane permeability assay.

The permeability of P. aeruginosa’s inner membrane in neutral versus mildly acidic medium was compared via the propidium iodide (PI) accumulation assay. The bacteria were harvested via centrifugation under the above-described conditions and resuspended in PBS containing PI (5 μM). The suspensions were incubated for 30 min, and the fluorescence was measured using SpectraMax M3 at the excitation wavelength of 535 nm and emission wavelength of 617 nm.

### Membrane potential microscopy and calculation.

The bacteria were labeled with a membrane potential-sensitive probe, 3,3′-diethyloxacarbocyanine iodide [DiOC_2_(3); Fluka], via the established protocol (62). Briefly, at the end of the logarithmic phase of growth, the bacterial cells (10^7^ CFU) were pelleted via centrifugation (Eppendorf 5810 R centrifuge; A-4-62 rotor) at 4°C and resuspended in PBS containing 150 μM of the probe, and the cells were incubated for 30 min at 37°C with 180 rpm shaking. As a control for dissipated membrane potential, a group of wild-type P. aeruginosa was treated with carbonyl cyanide *m*-chlorophenyl hydrazone (CCCP) at 5 μM. For fluorescence microscopy, a noncoated 8-well ibidi microscopy chamber containing the bacterial suspension was mounted on an LSM980 multiphoton microscope (Zeiss, Germany). The microscope captured green and red fluorescence (emissions at 505 and 670 nm, respectively) upon excitation at 488 nm. The images were taken with a 63× oil immersion objective at a resolution of 1,024 by 1,024 pixels. Additionally, the intensities of fluorescence emission in the green and red channels were measured along the individual bacteria using ZEN 2.6 (blue edition) software, and the red/green emission was calculated.

### Data availability.

All the information related to this study is included in this published article and its supplemental files.
