# Supplementary material for: A Mildly Acidic Environment Alters Pseudomonas aeruginosa Virulence and Causes Remodeling of the Bacterial Surface
Source: Microbiol Spectr. 2023 Jun 6;11(4):e04832-22. doi: 10.1128/spectrum.04832-22 (PMC10433952; doi:10.1128/spectrum.04832-22)
Supplement: Supplemental file 1 — Supplemental material. Download spectrum.04832-22-s0001.pdf, PDF file, 0.4 MB [file spectrum.04832-22-s0001.pdf]

Supplemental material

A mildly acidic environment alters *Pseudomonas aeruginosa* virulence and causes remodeling of the bacterial surface

Negar Mozaheb[1], Paria Rasouli[1], Mandeep Kaur[1], Patrick Van Der Smissen[2], Gerald Larrouy-Maumus[3], Marie-Paule Mingeot-Leclercq[1#]

1. Université catholique de Louvain, Louvain Drug Research Institute, Cellular & Molecular Pharmacology Unit (FACM), Brussels, Belgium.

2. Université catholique de Louvain, de Duve Institute, CELL Unit and PICT Platform, Brussels, Belgium.

3. Imperial College London, Department of Life Sciences, MRC Centre for Molecular Bacteriology and Infection, Faculty of Natural Science, London, United Kingdom.

#Address correspondence to marie-paule.mingeot@uclouvain.be

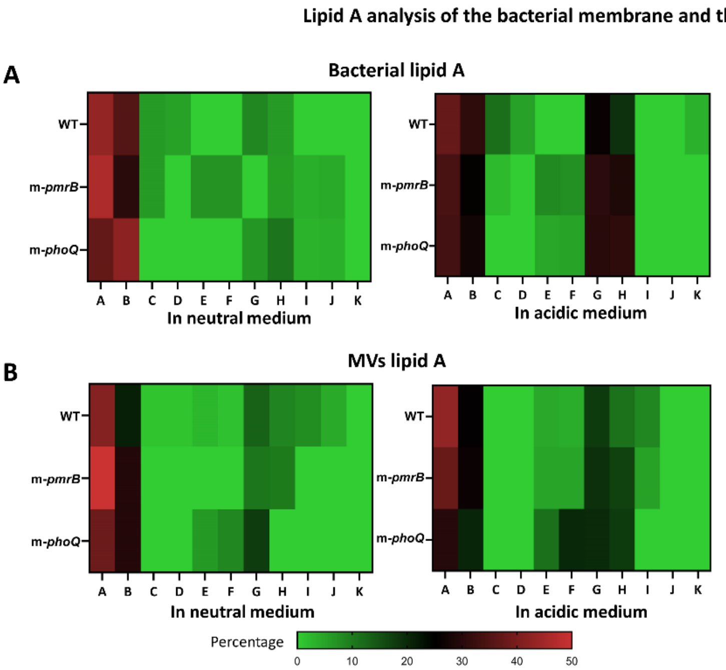

A	Penta-acylated, missing C10 acyl chain at position 3 (Double phosphorylated)
B	Hydroxylation of **A**
C	**A** structure including 1(L-Ara4N)
D	Hydroxylation of **C**
E	Hexa-acylated, lipid A (Mono phosphorylated)
F	Hydroxylation of **E**
G	Hexa-acylated, including C10 acyl chain at position 3 (Mono phosphorylated)
H	Hydroxylation of **G**
I	Hexa-acylated lipid A, including a C16:0 (palmitoyl) chain
J	Hydroxylation of **I**
K	**I** structure including 1(L-Ara4N)

Figure S1 Lipid A analysis. Comparative lipid A analysis of A) P. aeruginosa membrane and its B) MVs. The percentage of each detected lipid A species has been calculated based on the ratio of the lipid A species among the sum of all detected species. C) Detected lipid A species in the membranes and the MVs samples.

Table S1 MIC (µg/mL)

Strain	Neutral LB		Mildly acidic LB	
	Polymyxin B	Colistin	Polymyxin B	Colistin
WT	1	2	2	2
m-*pmrB*	0.5	1	1	2
m-*phoQ*	0.5	1	1	2

Growth curves and doubling times

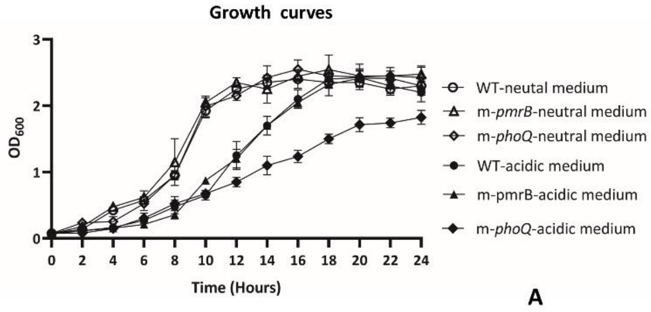

Doubling times

Strain	Neutral medium (min)	Acidic medium (min)
**WT**	44.3±6.2	49.6±1.1*
**m-*pmrB***	43.5±2.1	51.3±2.5**
**m-*phoQ***	48.2±1.2	59.0±4.2***

B

*Figure S2 Growth curve and doubling time analysis. A) strains growth curves over 24 hours. B) Comparative doubling time calculation during the logarithmic growth phase; the statistical comparisons are between the doubling times of the strains in the neutral medium and those in the acidic medium. Data presented as mean ± SD. Statistical analysis was performed on three biological replicates via a multiple t-test. ***: p <0.001, **: p <0.01 ,*: p <0.05*

Confirmation of the transposon mutants

After cultivating the stains on LB-agar plates in three replicates, we picked more than 10 colonies of each strain and mixed them in 10 mL of PBS. We used primers designed to amplify the gene from upstream or downstream of flanking regions of the genes of interest, paired with a transposon-specific primer (Table S2). The PCR program used was; 94 ◦C for 5min, 35 cycles at 94 ◦C for 1 min, 60 ◦C for 1 min, 72 ◦C for 1 min, and a final extension at 72 ◦C for 5 min.
The PCR products were analyzed *via* agarose gel electrophoresis. The colonies of transposon mutants contain an amplicon of about ~600 bp, whereas this was not the case for the wild-type colonies [1, 2].

Table S2 Primer sequences for confirmation of transposon mutants

Strain	Strain reference name	Primer pairs	
		Flanking primer	Transposon primer
m-*pmrB*	PW9024	GATACCGTGGAATGGGTGAC	CGGGTGCAGTAATATCGCCCT
m-*phoQ*	PW3131	GTTGAATTCGAAGTCCTCGC	GGGTAACGCCAGGGTTTTCC

Table S3 Primer sequences for qPCR

Gene name	Forward primer sequence (5' to 3')	Reverse primer sequence (5' to 3')	Reference
pmrA	AATACTGCTGGCCGAGGAC	GTCACCCATTCCACGGTATC	This study
pmrB	CGATCTTCACCCGCTTCTAC	AAGTGCAGTTCGACGATGC	This study
phoP	TGCGCCACCACCTCTATAC	GTATTCGCTGACCCGGTAGA	This study
phoQ	CCTGCTGGAGAACGCCTAT	GACCCACAGCTCCGAGTAAC	This study
arnT	CTTCTTCTGGCACGAACACA	AGCAACGGCAGGTAGAACC	This study
pagP	TACCTGCAGACCAGCGTCTA	GACCGGGTAGCTGTCACTGT	This study
pagL	GACAAGAGCTGGTGGCAGA	ATCGCCACCTTCCCAATAG	This study
pqsE	TGATGACCTGTGCCTGTTGG	GGCTGATCCCTCCTTCAACC	[3]
rhlA	CCTGGCCGAACATTTCAACG	TTTCCACCTCGTCGTCCTTG	[3]
algU	AGGATGCGGAGTTCTTCG	AACTGCTGGATGGTCTGG	This study
mucD	TAGCCTGCCGAACGAGAG	CGAGTTGATGCCGACCAC	This study
16SrRNA	AAGCAACGCGAAGAACCTTA	CACCGGCAGTCTCCTTAGAG	[3]

References:

1. Held, K., et al., *Sequence-verified two-allele transposon mutant library for Pseudomonas aeruginosa PAO1.* Journal of bacteriology, 2012. **194**(23): p. 6387-6389.
2. Jacobs, M.A., et al., *Comprehensive transposon mutant library of Pseudomonas aeruginosa.* Proceedings of the National Academy of Sciences, 2003. **100**(24): p. 14339-14344.
3. Wang, G., et al., *The membrane-active polyaminoisoprenyl compound NV716 re-sensitizes Pseudomonas aeruginosa to antibiotics and reduces bacterial virulence.* Communications biology, 2022. **5**(1): p. 1-17.
